# Large-scale analysis of high-speed atomic force microscopy data sets using adaptive image processing

**DOI:** 10.3762/bjnano.3.84

**Published:** 2012-11-13

**Authors:** Blake W Erickson, Séverine Coquoz, Jonathan D Adams, Daniel J Burns, Georg E Fantner

**Affiliations:** 1 Laboratory for Bio- and Nano-Instrumentation, École Polytechnique Fédérale de Lausanne, Batiment BM 3109 Station 17, 1015 Lausanne, Switzerland; 2 Mechatronics Laboratory, Department of Mechanical Engineering, Massachusetts Institute of Technology, Cambridge, MA 02139, United States of America

**Keywords:** adaptive algorithm, artifact correction, atomic force microscopy, high-speed atomic force microscope, image processing

## Abstract

Modern high-speed atomic force microscopes generate significant quantities of data in a short amount of time. Each image in the sequence has to be processed quickly and accurately in order to obtain a true representation of the sample and its changes over time. This paper presents an automated, adaptive algorithm for the required processing of AFM images. The algorithm adaptively corrects for both common one-dimensional distortions as well as the most common two-dimensional distortions. This method uses an iterative thresholded processing algorithm for rapid and accurate separation of background and surface topography. This separation prevents artificial bias from topographic features and ensures the best possible coherence between the different images in a sequence. This method is equally applicable to all channels of AFM data, and can process images in seconds.

## Introduction

Atomic force microscopes (AFMs) are a useful tool for investigating nanoscale surfaces. They have applications in physics, materials science, chemistry, biology and nanotechnology. AFMs generate detailed three-dimensional images of surfaces with nanometer and subnanometer resolution [1–12]. The raw imaging data has to be post-processed to eliminate artifacts arising from distortions inherent in the technique or a specific instrument. Sample tilt, scanner bow and other artifacts corrupt the true topography. Removal of these artifacts is requisite to obtain the true topography of a sample [13–17]. In most AFM studies, the goal is to generate a single image of a sample surface or surfaces of multiple samples. The resulting data sets are relatively small and easy to correct by hand. In an emerging part of the field, the goal is no longer to see just the detailed surface topography; but rather, to also see how this topography changes as a function of time or treatment [18–24]. High-speed AFM (HS-AFM) focuses on reducing imaging time to track faster dynamics than can be observed with existing instruments [25–29]. Where conventional AFM generates only a few images, HS-AFM generates hundreds of images. This amount of data creates a significant increase in the amount of image processing needed to extract the true sample topography, which can no longer be performed by the user on an image-by-image basis [15,17]. In HS-AFM, there is a real need for an automated processing routine that optimizes the image processing for each image without losing essential topographic information. One of the most important considerations in automating processing is that it must not introduce processing artifacts, and it must maintain coherence between images in a dataset to allow for rapid comparisons between frames [30–31]. Automated, AFM quality-control check in semiconductors and other nanotechnologies [32–34] also creates large data sets, which would also benefit from reliable automated image processing. In order to automate the image processing, it is important to define a metric by which the success of the image processing can be judged, and for which the processing is optimized. In the next sections we will evaluate the source and effect of typical AFM image distortions and derive a suitable optimization metric.

### Sources of inherent distortions

**Figure 1:**
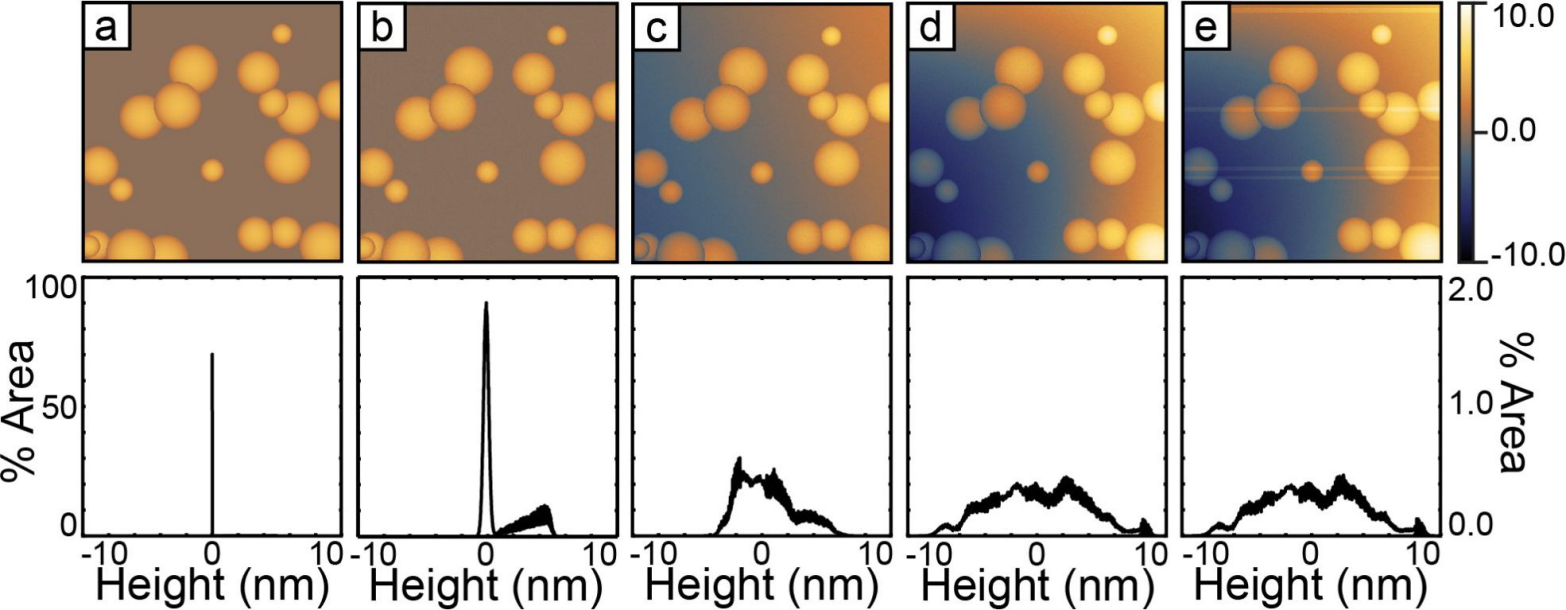
This figure shows the cumulative effects of typical distortions on model AFM data. Panels A though E show a sample with arbitrary topographic features (randomly placed and sized half spheres) and the corresponding histograms below. The first column shows the pure topography. The second column shows the addition of random noise. The third column shows the effects of an arbitrary sample tilt. The fourth column shows the effects of an additional second-order polynomial representing scanner bow. The fifth column adds arbitrary 1-D offsets in the fast-scan direction. Each artifact successively broadens the peaks in the histogram. The histogram in column A uses the vertical scale on the left. The histograms columns B through E share a common vertical scale shown on the right. All images share a common color scale shown on the right. All the histograms are plotted over this same range.

All AFM images will contain artifacts in the *x*–*y*-direction as well as the *z*-direction. This work will address only the distortions found in the *z*-direction. There are two fundamental classes of image distortions in the *z*-direction, i.e., 2-D distortions and 1-D distortions. Both of these distortions are well-known and have been commented on since the earliest prototypes. 2-D distortions manifest over the whole image and deform 2-D surfaces into 3-D. The most common example of this is sample tilt. Tilting the sample normal relative to the scanner normal gives the entire image an apparent tilt. Piezo tube scanners (which are often used in AFM instruments) also generate inherent distortions in the image, creating an additional bow in the apparent topography [13]. Some of the early work in the field addresses the need to correct these background distortions appropriately by fitting only the background regions to a polynomial of the correct order [35]. 1-D distortions cause relative offsets from scan line to scan line. The sources of these distortions include laser-mode hopping and changes in the tip–sample interaction. These 1-D distortions cause apparent discontinuities in the topography of the sample, but do not represent an actual topographic feature. Most importantly, 1-D distortions generally appear as offsets in the *z*-data. Potential corrections for these types of artifacts have been addressed well in a direct fashion from starting principals by Starink and Jovin [35], and again in a less direct fashion that has some potential pitfalls with an emphasis on biological samples [36]. Figure 1 shows how these types of artifacts distort an artificial image along with corresponding histograms. In Figure 1A, half-spheres of arbitrary size were placed randomly within the image. A histogram of the *z*-heights in the image shows a spike at the background level, and a distribution of heights from the sphere (along the axis *x*-axis). Figure 1B shows the addition of a small amount of random noise added in the *z*-direction. This noise leads to broadening of the histogram peak. The peak now appears Gaussian with a small distribution. Figure 1C imparts a small tilt to the sample (less than 10 nm over 3 µm in either direction, which is very good for most samples). The background peak has been broadened so far as to be nearly indistinguishable. Figure 1D adds a second-order polynomial distortion to simulate scanner bow (less than 10 nm over the scan range, which is well within reason for most AFMs). The peak is no longer visible in the histogram. Finally, Figure 1E adds random vertical offsets to some of the scan lines. These offsets simulate the sorts of line skips commonly observed while imaging. As can be seen from the histograms, each successive distortion broadens the peak in the histogram. We will, therefore, test the suitability of calculating the distribution of the histogram as a metric for judging the success of automated-image-processing steps.

### Basic requirements for successful image correction

The goal of the AFM image-processing algorithm described in this paper is to remove imaging artifacts and generate the best possible representation of the true sample topography. The achievement of this goal has one basic prerequisite, an area that can be used as a reference geometry. In most cases, this will be an area that can be considered as flat in the true topography of the sample. The image-processing algorithm then attempts to extract the global distortions in the image from observations of the flat reference region. These global distortions are subtracted from the complete image. In practice, this criterion is often met in AFM images, since the sample preparation generally utilizes flat substrates, such as mica or silicon wafers. In the case of semiconductor processing, the reference geometry could also be a specific area of the processed device that is flat or has a known shape. For the purpose of this work, we use the assumption that there is some part in the image that corresponds to a flat background, since this is the most common case. The algorithm automatically determines this background region and performs the necessary background correction. The algorithm uses minimization of the standard deviation of the image histogram to judge the success of the background detection and the image processing.

### Image term description

Throughout the rest of this paper we will use the following conventions when referring to images for the sake of clarity. First, an image is defined as an array of scan lines along the slow scan axis,

[1]
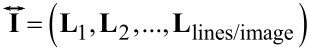


where each scan line is an array of pixels in the image along the fast scan axis.

[2]
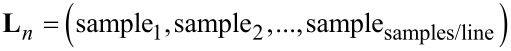


### Standard deviation as a metric for image flatness

In order to show that the standard deviation of an image is a suitable metric for monitoring the progress of image flattening we can use uncertainty propagation. We can start by describing the AFM image, 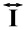, as the sum of two height fields, the sample topography, 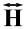, and the inherent geometric distortions, 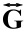:

[3]
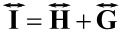


The square variance of the image can be written as:

[4]
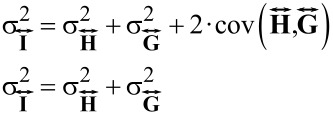


The equation simplifies to the final form because the covariance of the two fields is zero (they are uncorrelated variables for the types of distortions described in this paper). This means that 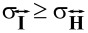; thus, the geometric distortions will always broaden the distribution of observed heights relative to the true sample topography. This also means that as long as the processing steps only affect 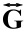 (flattening is only performed on truly flat regions, and not real topography), the minimization of 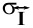 will improve the image.

### Commonly used methods for correction of inherent distortions

**Figure 2:**
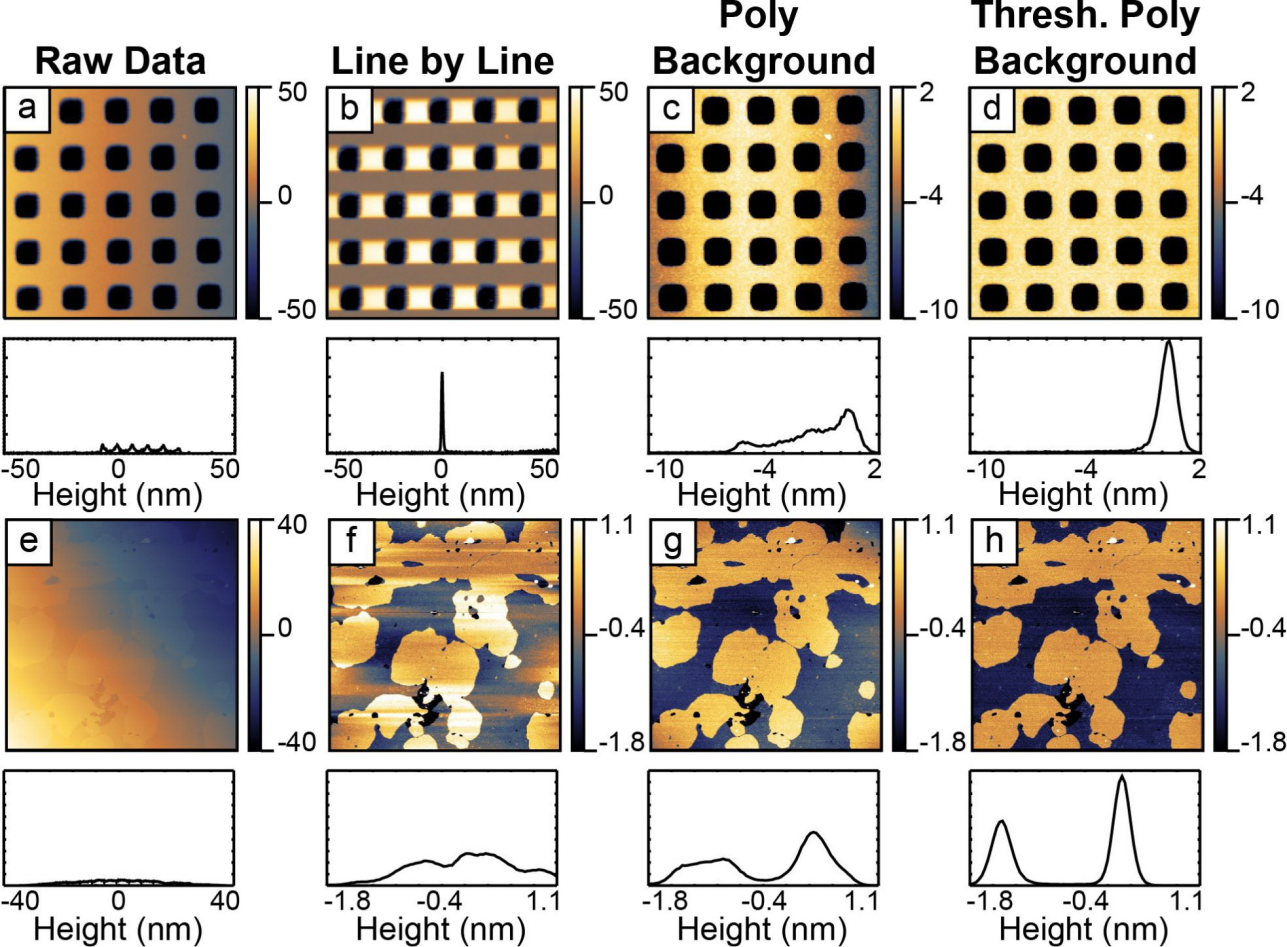
Panel A shows the raw data of a calibration grating. Panel B shows the result of line-by-line polynomial subtraction on the grating. Panel C shows global 2-D polynomial subtraction. Panel D shows a thresholded 2-D polynomial subtraction excluding the pits from the polynomial fit before subtraction. The histograms of each image are shown below. The vertical axis is the same for each histogram. In both the images, and the histograms, the scale has been focused on the upper portion of the grid in order to highlight the distortions in the image. Panel E shows the raw data of a mixed lipid bilayer on mica. Panel F shows the results of line-by-line second-order polynomial subtraction. Panel G shows 1-D artifact correction followed by 2-D second-order polynomial subtraction. Panel H shows the results of 1-D artifact correction followed by 2-D second-order polynomial subtraction by using the highest lipid domain to calculate the background for removal. The histograms of each image are shown below. The vertical axis is the same for each histogram. The median of each panel has been set to zero to allow visual comparisons. Panels D and H both show improvements in the representation of the surface in comparison to the other results. These improvements are shown clearly in the histograms. The grid was imaged under tapping mode in air. The lipid sample is the height data from QNM mode in fluid. The vertical scales of each image are in nanometers.

The goal of AFM image processing is to correct the inherent distortions mentioned above and recover an accurate representation of the undistorted surface topography. A simple, often used, method is 1-D line fitting, which is an effective way to get a rough representation of the surface by removing much of the scanner tilt and bow at the expense of some induced artifacts. Methods for the subtraction of 2-D distortions, which add less artifacts than does 1-D line fitting, are 2-D polynomial fits. 2-D polynomial fits can be performed by using either the whole figure for the fit, or only certain regions of the image determined by using thresholds. Figure 2 shows a comparison of different processing methods on a standard calibration grating as well as on a lipid bilayer of mixed composition. Figure 2A and Figure 2E show the starting data. If present, the 1-D errors must be corrected first because any attempts to correct for the 2-D distortions will be biased by these 1-D offsets. To do this, the offset caused by the 1-D distortions is removed from each line. Higher order 1-D operations, such as line fits or higher order polynomial subtraction, should not be used to correct the inherent 2-D distortions because they will destroy interline relationships in the data, and can generate false artifacts between lines [13,31,37], see Figure 2B and Figure 2F which show the results of 1-D second-order polynomial removal. This line-by-line polynomial subtraction generates many artifacts in the data, for example the surface surrounding the pits appears raised along the fast scan axis in Figure 2B, and continuous levels in Figure 2F have offsets from line to line and are not perpendicular to the image plane. Figure 2C and Figure 2G show the removal of 1-D offsets followed by a second-order background polynomial removal. For the grid, there is a significant improvement in the representation of the surface. In Figure 2F, the sample topography is of the same order as the 2-D distortions. While 2-D operations are less prone to induce artifacts, performing a global 2-D polynomial fit and background subtraction leaves significant residual distortions (Figure 2G). These distortions can be avoided by using a thresholded flattening, instead of a global flattening Figure 2D and Figure 2H (details discussed in Section Results and Discussion, “Algorithm description”). We conclude that a suitable way to process the images is to first determine and subtract the line-by-line offsets. Second, fit only the part of the image that is *the flat background* with a 2-D polynomial. Finally, subtract the calculated 2-D polynomial from the entire image. For an automated algorithm, the problem reduces to accurately distinguishing *the flat background* from the sample topography. In the rest of this paper, we describe a method for iteratively determining both *the flat background*, and the line-by-line offsets. Once these quantities are known, subtracting the line offsets and correcting the 2-D distortions can be performed with only two image-processing steps on the raw data.

## Results and Discussion

### 1 Algorithm description

**Figure 3:**
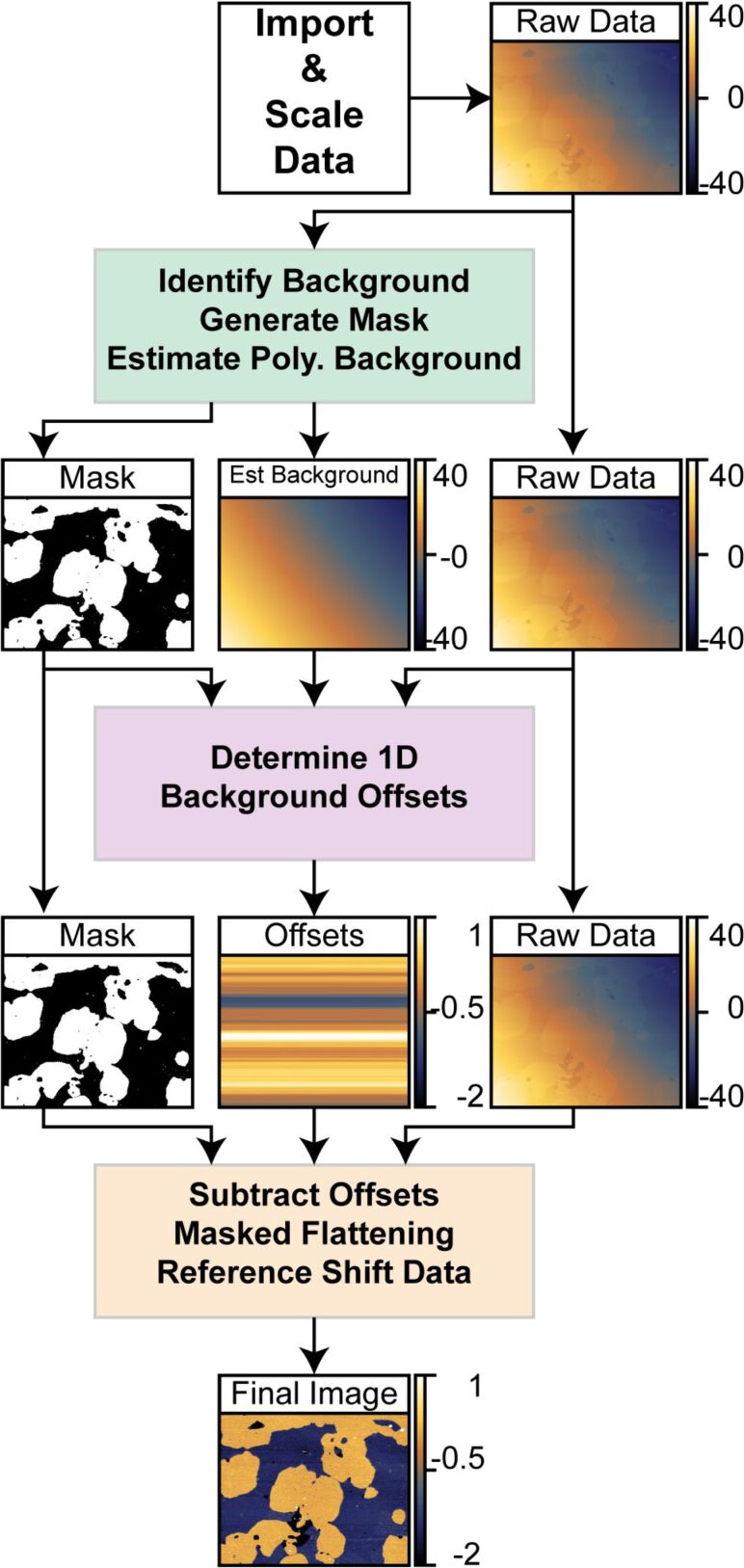
This figure outlines how an image is corrected automatically. There are three main operational blocks used in the process. Each block starts with the raw data as input and generates unique outputs. The first major block applies a series of 1-D offset corrections and thresholded polynomial background subtractions to generate a masked image of the region to be flattened as well as an estimate of the polynomial background. The second block subtracts this estimated background from the raw data and runs thorough a series of median corrections and flattening steps only on the data within the masks. This sequence determines the unique 1-D offset for each line. The third block subtracts these 1-D offsets from the raw data and performs a single polynomial fitting on the data within the mask. This background is subtracted from the entire image (raw-offsets). Finally, the height of the background is shifted to a defined reference value to ensure continuity within the image sequence. All vertical scales in the figure are in nanometers.

Figure 3 shows a general flow diagram of the processing algorithm from data import to final output. The algorithm has three major blocks: (a) **identify the background** region, **generate a mask** of the background region and **estimate the polynomial background**; (b) **determine 1-D background offsets** from the raw data within the mask; and (c) **subtract 1-D offsets** from the raw data followed by a single **masked background flattening**. This final step ensures that a minimal number of modifications are performed on the raw data to generate the final output. The final output is offset to consistent values and exported for later use. The results of the automated processing routine on an example lipid bilayer of mixed composition are shown as the inputs and outputs of each major block. Each block will be discussed separately in detail.

#### 1.1 Identify the background, generate a mask, estimate the polynomial background

The purpose of this section is to identify the background region in the image and estimate the initial polynomial background. In order to accomplish this, the algorithm does the following: (a) roughly corrects 1-D offsets present in the image (scars and offsets); (b) monitors the distribution of height values in the image to track the progress of each step, and rejects steps that broaden the distribution; (c) intelligently removes global tilt and scanner bow (often greater than sample topography); (d) adaptively identifies the background; (e) fits 2-D polynomials to the identified background; and (f) removes these fitted polynomials from the image.

**Figure 4:**
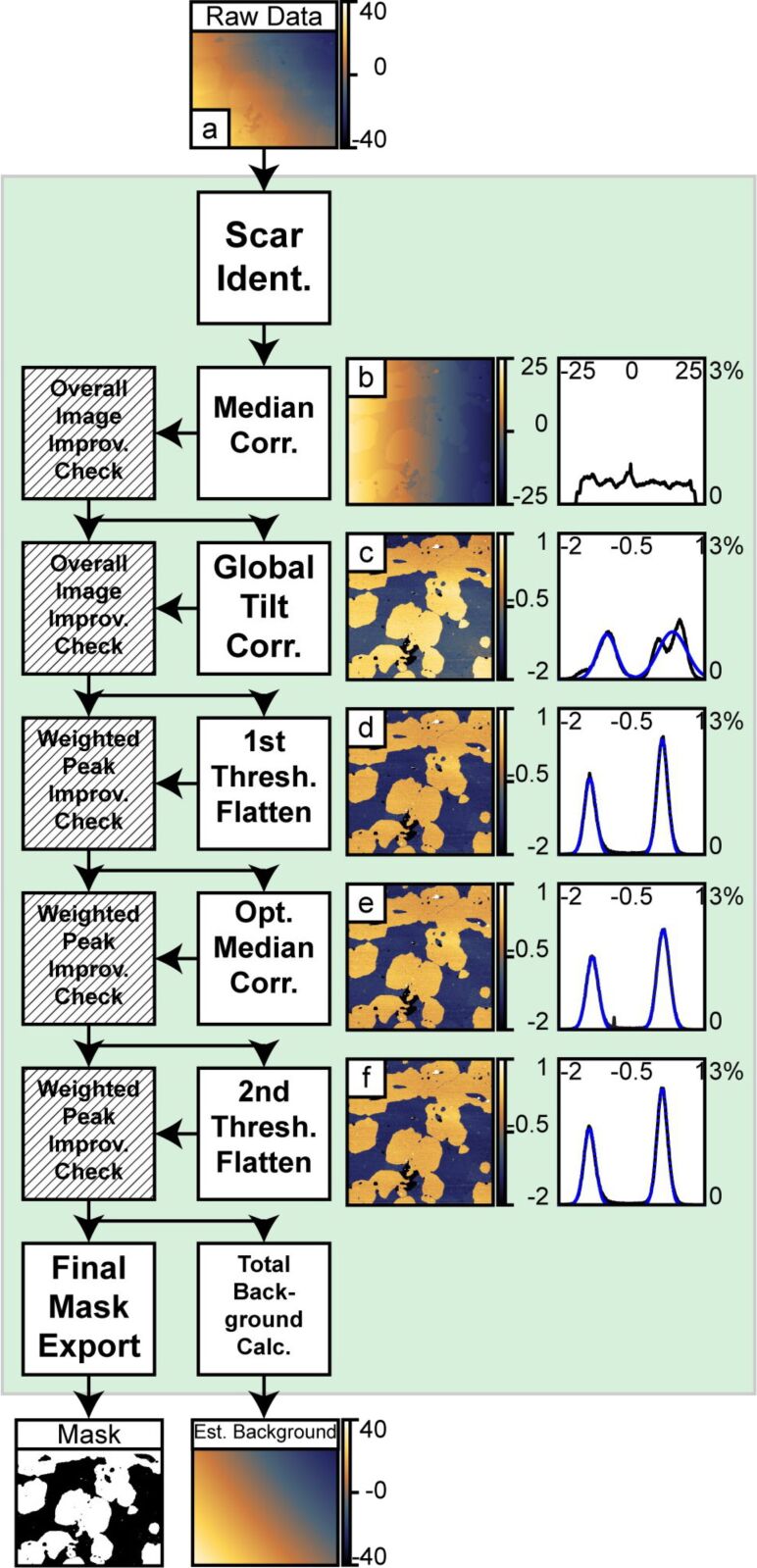
This figure shows the structure of the processing algorithms for identifying the region to be flattened and estimating the background. An initial scar detection followed by median correction and a median-difference correction is applied. This corrects for line skips in the source image. Following the 1-D artifact correction, a global correction is applied. The primary function is to remove overall sample tilt and some second-order distortions. The image is then adaptively thresholded and a 2-D polynomial is subtracted. After this step there is an optional second median difference correction to correct discontinuities missed in the previous median-difference correction. A second round of thresholded processing corrects for residual image distortions. After each step that manipulates data, i.e., the first median correction through to the final thresholded processing, a weighted improvement check ensures that the step had improved the image. In the case when a step degrades the image, the algorithm passes the previous result. The final mask generated after the last successful step as well as the sum background of all successful flattening steps is passed on to the next block of the algorithm. The right half of the figure shows the corresponding images and histograms for each step. Gaussian fits to the two distinct levels are shown with dotted blue curves. The AFM data in this figure was collected in QNM mode in fluid. The vertical scale in all images and the horizontal scale in all histograms is in nanometers.

**1.1.1 Scar identification and median correction**: The first process is to import the data from the binary format and convert it into relevant units. The standard deviation of the image is calculated as a reference point. Figure 4A shows the data as captured. Sample tilt dominates the image.

A basic scar identification and correction step is used to minimize the influence of the scars on the correction of 1-D artifacts. Scars are identified by detecting sections within a line **L***_n_* that are greater than surrounding lines by more than two times the standard deviation of the difference in medians. (Equation 5). Then each data point identified within a scar is replaced with the median of five neighboring data points in the last line before the scar:

[5]
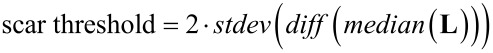


*diff*(**X**) calculates differences between adjacent elements of **X**. If **X** is an array, then *diff*(**X**) returns an array, one element shorter than **X**, of differences between adjacent elements: [**X**_2_ **− X**_1_, **X**_3_ − **X**_2_,…,**X***_n_* − **X***_n_*_ − 1_] of all lines in the image.

1-D artifacts, which introduce relative offsets, are corrected for by using *median corrections*. First, a line-by-line median-offset correction is applied to all lines. For each scan line, **L***_n_*, the median of the scan line is subtracted from all the values in that scan line (Equation 6).

[6]
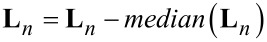


Second, a weighted median-difference correction corrects artifacts from the median correction caused by differences in topography in the image. This process is only performed on lines not previously identified as scars. The median difference correction calculates the median of the difference between two consecutive scan lines excluding outliers greater than two times the standard deviation of the differences between scan lines. This outlier exclusion reduces influence from edges within a scan line. The median difference value is subtracted from all the values within the second scan line, see Equation 7.

[7]
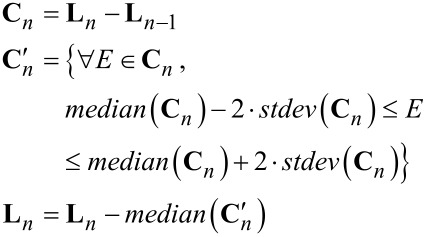


Third, an additional iterative refinement minimizes residual offsets generated from changes in topography. This subtracts a running average of the median difference between the scan line and up to the preceding *k* scan lines within a defined threshold. This does two things: one, it further excludes edge effects along topographic contours; and, two, it maintains continuity over long distances in the image and prevents the build up of high-frequency errors from scan line to scan line. This process runs iteratively in alternating directions. Currently, the algorithm uses four iterations with a maximum span, *k*, equal to 15 lines.

[8]
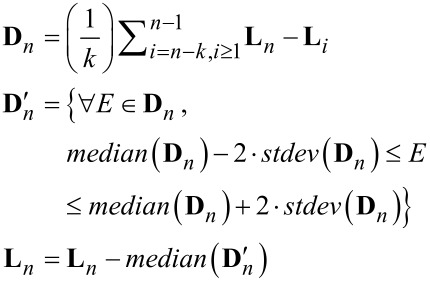


These steps handle discontinuities in the original image that do not represent actual topography. In cases where there is significant topographic variation parallel to the fast scan axis, i.e., a calibration structure, these corrections can actually introduce significant artifacts into the data and should not be used. In these cases, these 1-D corrections can be turned off in the algorithm. In Figure 4B, the initial median correction improves the image by removing both the 1-D artifacts as well as most of the tilt in the vertical direction. It is important to note, that in all these operations, there is always only a constant offset subtracted from all points in a line, and not a linear fit or higher polynomial. Such higher-order 1-D corrections can create artifacts that cannot be undone at a later stage.

**1.1.2 2-D Tilt removal**: The third process is a basic polynomial surface fit of either first or second order and subsequent removal to facilitate the thresholding process by removing enough distortion to make it possible to separate topographic features from the background. Primarily, this removes sample tilt, which has a significant contribution to the variation in the observed heights. In cases of very flat samples where the changes of the topography are on the same order or smaller than the sample tilt or scanner bow, removal of a second-order surface generally improves the separation of the topography from the background. The algorithm does a separate first-order and second-order removal process and calculates the standard deviation of both results. The standard deviations are compared to the standard deviation after the median correction. The image with the lowest standard deviation progresses to the next step. Figure 4C shows the results of the first, unthresholded background correction. A second-order polynomial removal improved the image the most. After this step, the two distinct levels in the system manifest in both the image and the corresponding histogram. Some residual tilt remains in the image, as the bottom is somewhat higher than the top.

**1.1.3 Thresholded flattening**: The fourth process is an adaptive image thresholding algorithm and background removal. In order to calculate the appropriate threshold for the 2-D background removal, the algorithm identifies discrete peaks in the histogram corresponding to different topographic levels in the image. In the absence of discrete levels, only one general peak is used. The histogram is fit to a summation of *m* ≤ 8 Gaussian functions centered at each peak, *z_i_*, where *i* ranges from 1 to *m*. In the case that the number of levels is greater than *m*, only *m* peaks with the greatest area will be fit. From the Gaussian fit, the standard deviations, σ*_i_*, and areas covered, *A_i_*, are calculated. Nominally, the threshold for the 2-D surface fitting is *z_j_* ± *n*σ*_j_*, where *n* is a user-defined range and *j* is the peak used for the 2-D surface fit. (The algorithm default is to use the highest peak found in the histogram. Based on prior knowledge of the sample, the user can override this default and specify which peak to fit.) Since this span can include contributions from other peaks, the actual span can be narrowed to the intersection of the *j*th peak with the next closest peak. All the data within the threshold is fit to a polynomial surface of a given order (up to fifth). The extrapolated background is subtracted from the whole image.

**1.1.4 Improvement check**: In order to test if a particular step has improved the image, the peak detection and Gaussian fit is repeated on a histogram of the corrected data. While the thresholded flattening only operates on a single level, the improvement check monitors the progress of all the fit peaks. This allows the algorithm to follow the progress of a sample with many levels. The criteria for this conditional check are:

1. The total area of the fits must not decrease by more than a small tolerance with respect to the image before the processing step.
2. A weighted average, 
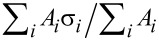
, of the standard deviations is computed both before and after. This term must not increase by more than a small threshold.

If either of these conditions fails, the algorithm rejects the process and passes the previous image to the next step. In the case of the initial background removal the whole image is used. After the initial background removal, only regions identified as independent levels are considered. The user defines the number of levels present in the system. In the case of the lipids or grids, both independent levels are considered; whereas, in the case of a random sample on a substrate, only the substrate would be considered. Figure 4D shows the results of the first thresholded processing. This results in a significant improvement of the overall image flatness, shown in both the image and histogram. The algorithm based the thresholding on the higher level since it had the greatest area.

#### 1.2 Determine 1-D background offsets

**Figure 5:**
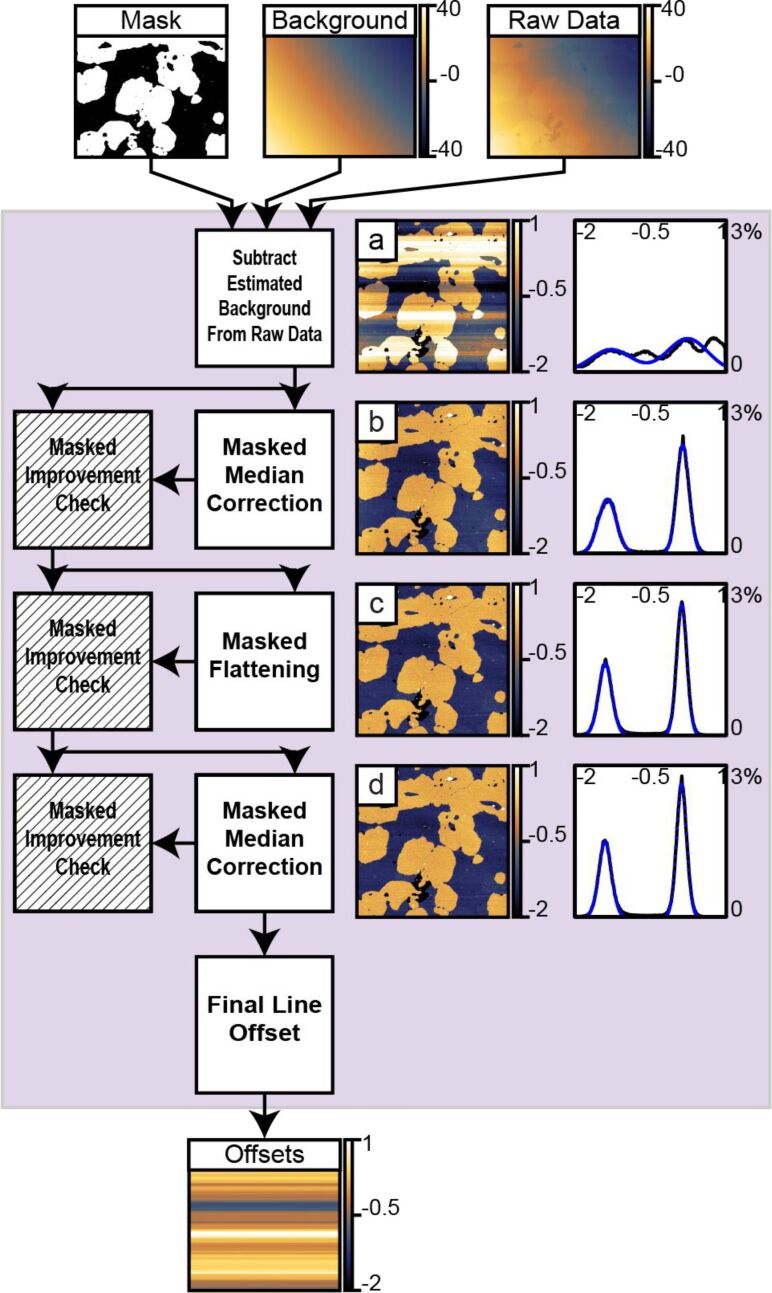
This figure shows the structure used to determine the 1-D offsets present in the image. First, the estimated background from the prior block is subtracted from the raw image. Second, a median correction is performed on the data within the mask. Third, an additional polynomial flattening on the masked region reduces the influence from the topography. Fourth, a second masked median correction refines the calculated offsets. After each step, except the initial background subtraction, an improvement check on the masked region verifies that the step improved the image. The final offsets are determined and passed through to the next section. The vertical scale of all images and horizontal scales of all histograms are in nanometers.

The purpose of this section is to accurately determine the 1-D offsets that exist from line to line. While the operations used above in Section 1.1 do a reasonable job, they are still prone to influence from sample topography. In order to minimize the influence of topography the algorithm performs its corrections only on the masked background region determined in Section 1.1. First, the estimated background from Section 1.1 is subtracted from the raw data in order to generate a reasonable starting point for the first masked median correction (Figure 5A). The masked median correction subtracts the median of all the data within the mask on a given line, from that line (Figure 5B).

[9]
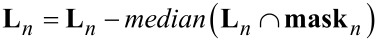


The second step is an additional polynomial flattening of the masked data in order to ensure a flat background (Figure 5C). This step is followed by an additional masked median correction (Figure 5D). Following each step, a check is performed to ensure that the step decreased the standard deviation of the data within the mask. Finally, the cumulative offsets are calculated for each line. In the case where a line has no pixels contained in the mask, the algorithm calculates the offset from the final output of Section 1.1 instead. These final outputs are passed on to the next section.

#### 1.3 Subtract offsets, masked flattening, reference shift data

**Figure 6:**
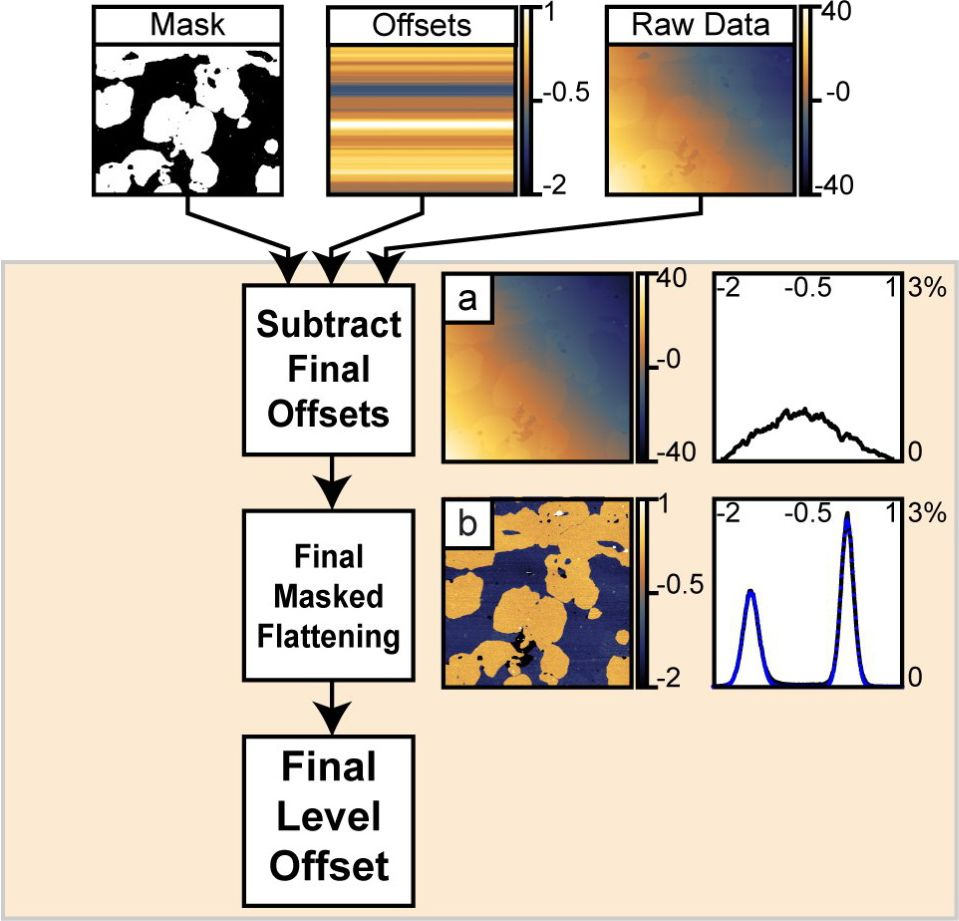
This figure shows the steps used for the final image correction. First, the 1-D offsets are subtracted from the raw image. Second, a final masked polynomial flattening is performed. Third, a reference offset is added to all data in the image prior to export. The vertical scale of all images and horizontal scales of all histograms are in nanometers.

This final section of the algorithm subtracts the offsets calculated in Section 1.2 from the raw data (Figure 6A). Next, the data within the mask calculated in Section 1.1 is fit to a single 2-D polynomial. The resulting polynomial is subtracted from all the data (Figure 6B). A final histogram is computed and fit with Gaussians. The difference between the defined background reference height and the fitted center of the background peak in the final histogram is added to all the data. This final data is exported for future use.

### 2 Processing high-speed dynamic images in biological systems

#### 2.1 HS-specific artifacts

Operating AFMs at high speeds has the potential to generate new distortions not normally observed at conventional speeds. Such artifacts include structural scanner resonances coupling into the sample topography. These sorts of spurious resonances can be observed in piezo tube scanners as a turn-around ripple in the images (damped oscillations of a given frequency). This stems from excitations in the lateral direction coupling into the vertical motion. Because the scanner used in these experiments has a relatively low structural resonance, this turn-around can be excited to a substantial extent at even moderate scan speeds. Given that the amplitude of the turnaround ripple in tube scanners like the one used in these experiments can easily be greater than the topography of the system, the turn-around ripple must be dealt with in some fashion. The best way is to avoid it altogether through either input shaping [38–41] of the drive signals or through electrical damping of the resonances [42]. For our experiments, we use a self optimizing method that determines the scanner resonances and compensates them with an input shaper [38]. Using this resonance-compensator system, the amplitude of the turn-around ripple in the image can be nearly eliminated in a fashion that is sample independent and completely transparent to the rest of the experiment. While this method works very well, some residual, subnanometer distortions can remain.

#### 2.2 Vertical median correction

**Figure 7:**
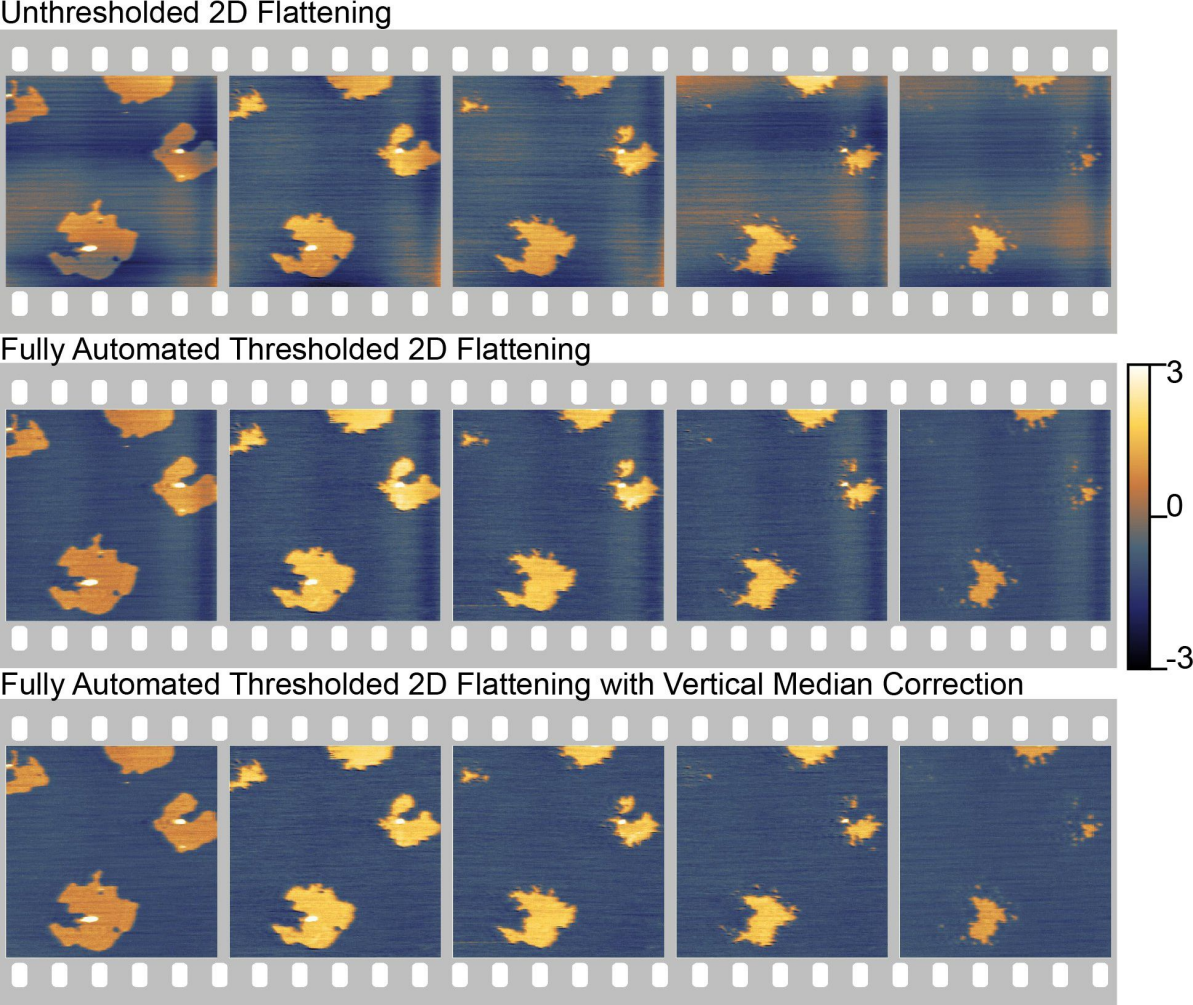
Comparison of unthresholded 2-D background removal to the full, iterative thresholded background removal for a sequence of HS-AFM data. The upper row shows the unthresholded result. The fully automated processing using the lower level for the background calculation is shown in the middle row. In the bottom row, an additional vertical median correction has been used. This corrects some of the residual artifacts from the turn-around ripple of the scanner, which are not corrected with the resonance compensator described by Burns et al. [38]. Each image is a 4 µm image with 256 lines captured at 78 lines/second while tapping in fluid. These imaging parameters give an approximate acquisition of 3 seconds per image. Each frame in this figure represents every third image in the overall sequence, approximately 9 seconds apart. (Full image sequence as Figure S1 in Supporting Information File 1 or in movie format as Supporting Information File 2). In the unthresholded sequence, many artifacts remain, making it difficult to track the progression. In the fully automated sequence, the automated algorithm does a much better job of correcting the data and outputting consistent images for comparison.

While model-based filters do a very good job of reducing the amplitude of the turn-around ripple, they may not completely eliminate it. This is especially problematic on very flat samples with nanometer scale topography, where a subnanometer distortion is a significant part of the overall topography, such as in the images of lipid membranes shown in Figure 7. This residual turn-around ripple can be seen on the right-hand side of the images in the middle row. The turn-around ripple appears on the right side of the image because retrace images are being shown. This residual error can be corrected by using an additional vertical median correction on the masked data after the final background subtraction in Section 1.3 and before the final offset. This vertical median correction is exactly the same as Equation 9, but it runs in the perpendicular direction. This correction is only applicable to HS-AFM data that exhibits some turn-around ripple and should not be used otherwise.

Figure 7 demonstrates the utility of the automated processing algorithm on a dynamic model biological system. The sample consists of two phase-segregated lipids, DLPC (blue) and DPPC (gold). The image sequence shows the rapid degradation of the DPPC domains following rapid heating from interactions with the AFM tip. The sequence shows every third image. The upper sequence shows the results of standard unthresholded flattening and the lower sequence shows the results of the fully automated processing. Both sequences use the same polynomial background orders. The fully automated processing does a much better job of identifying the background and flattening it correctly. This makes it much easier to follow the changes in the system. Moreover, this automated flattening process allowed for a significant improvement in the speed of processing without sacrificing the quality of the results. The images shown in Figure 7 and Figure S1 in Supporting Information File 1 are only a few sample images from a much longer image sequence many hundreds of images long. The entire sequence was processed in only a few hours, representing a significant time saving compared to hand-correcting each image.

## Conclusion

We have developed an automated, adaptive image-processing algorithm for high-speed AFM image sequences. This is achieved by identifying the background region and determining line-by-line offsets using an iterative process. The output of this iterative process is used to perform a single line-by-line offset correction, followed by a single 2-D polynomial-background removal step. This order of operations ensures that a minimal amount of manipulation is performed on the raw data to generate the final output. During the iterative background-identification process, the algorithm uses the standard deviation of one or more peaks in the height histogram of the image as a metric for determining the accuracy of the thresholded background identification. A similar metric is used to determine the accuracy of the line-by-line offset correction. The algorithm is specifically tailored to image sequences, ensuring consistent processing, offsets and contrast settings between frames in AFM movies in a short time period. On a standard PC, each channel takes about 10 seconds to process. This algorithm can be used on AFM data from most modes. We have demonstrated its applicability to data acquired in tapping mode in air, tapping mode in fluid, and quantitative nanomechanical mapping (QNM) in fluid. Finally, this algorithm can also be applied to strongly stepped samples, such as atomic layers. Figure S1 in Supporting Information File 1 shows the correction of samples with four and eight distinct levels. In both cases, the error is less than the noise in the image. With the release of modern HS-AFM systems by commercial manufacturers, this sort of automated processing will provide a significant benefit to this emerging research area in surface science and related fields. The algorithm and an associated graphical user interface are available at http://lbni.epfl.ch under the software section.

## Experimental

### Lipid preparation

Small unilamelar vesicle mixtures of 1,2-dilauroyl-*sn*-glycero-3-phosphocholine (DLPC) and 1,2-dipalmitoyl-*sn*-glycero-3-phosphocholine (DPPC) were prepared by sonication. Both lipid types were purchased from Avanti Polar Lipids Incorporated (Alabaster, AL, USA). Lipid powders were mixed prior to vesicle formation at a nominal molar ratio of 1:2, DLPC/DPPC. Vesicle solutions at 1 mg/mL were formed by transferring an appropriate mass of lipid into glass vials and dissolved with chloroform. The chloroform was evaporated off with dry nitrogen gas, leaving a thin film on the glass vial. The film was hydrated with Milli-Q water (Millipore, Billerica, MA, USA), generating large multilaminar vesicles (LMVs). The LMVs were then sonicated with a probe sonicator (BioLogics Inc, Manassas, VA, USA) to generate small unilaminar vesicles (SUVs). The SUVs were centrifuged to remove metal particles left from the probe sonicator. A 35 µL amount of the lipid preparation was warmed to 37 °C and deposited onto freshly cleaved mica surfaces, forming bilayers through vesicle fusion. Surfaces were allowed to incubate for at least a half hour in a humid environment at room temperature.

### AFM imaging in air

Images were captured on a Multimode system with an E-scanner (Bruker Nano: Santa Barbara, CA, USA). Standard TESPA (Bruker AFM Probes: Camarillo, CA, USA) tapping cantilevers were used. The cantilever was driven at 348.44 kHz with an amplitude of 20.14 mV. Images at a size of 14.25 µm were captured at 512 × 512 pixels with a line rate of 1.5 lines/second. The data shown in the paper are crops from the center of the acquired image.

### Quantitative Nanomechanical Mapping (QNM) – AFM imaging in fluid

Images were captured on a Multimode system with an E-scanner (Bruker Nano: Santa Barbara, CA, USA). A standard DNP-A (Bruker AFM Probes: Camarillo, CA, USA) cantilever was used with a spring constant of 0.40 N/m. Images at 5 µm were captured at 512 × 512 pixels with a line rate of 1 Hz. Manual control of the QNM parameters was used to minimize the applied force on the sample and the QNM drive amplitude. Each channel’s acquired limits were minimized to limit bit quantization in the DSP.

### HS-AFM imaging in fluid

Images were captured on a modified Multimode system with an E-scanner (Bruker Nano: Santa Barbara, CA, USA). A customized small-lever head allowed for the use of small cantilevers (SCL-Sensor.Tech., Vienna, Austria). The cantilever had a resonance frequency in fluid of 266.49 kHz, a spring constant of 0.54 N/m and a Q value of 2.68. Square areas of 4 µm were scanned at 78 lines/second at 256 × 256 pixels giving an approximate image acquisition time of 3 seconds/image. The fast-scan drive signal was passed through a custom filter designed to minimize the excitation of the tube scanner resonance [38]. The internal PID feedback of the Nanoscope 5 controller was bypassed with a Labview controlled FPGA based PID with a loop rate of 575 kHz.

## Supporting Information

File 1 Further details on imaging and image processing

File 2 Image sequence movie
The movie shows the entire image sequence of the fully corrected data, with vertical median correction, from Figure 7. Each frame is approximately three seconds apart. The vertical scale of all images is in nanometers.

File 3 User Manual
The user manual presented here contains a brief description of how to use the program and the parameters available for each channel.
