# Supplementary material for: Large-scale analysis of high-speed atomic force microscopy data sets using adaptive image processing
Source: Beilstein J Nanotechnol. 2012 Nov 13;3:747–58. doi: 10.3762/bjnano.3.84 (PMC3512124; doi:10.3762/bjnano.3.84)
Supplement: File 1 — Further details on imaging and image processing [file Beilstein_J_Nanotechnol-03-747-s001.pdf]

Supporting Information

for

Large-scale analysis of high-speed atomic force microscopy data sets using adaptive image processing

Blake W. Erickson[1], Séverine Coquoz[1], Jonathan D. Adams[1], Daniel J. Burns[2] and Georg E. Fantner*[1]

Address: [1]Laboratory for Bio- and Nano-Instrumentation, École Polytechnique Fédérale de Lausanne, Batiment BM 3109 Station 17, 1015 Lausanne, Switzerland and [2]Mechatronics Laboratory, Department of Mechanical Engineering, Massachusetts Institute of Technology, Cambridge, MA 02139, United States of America

Email: Blake W. Erickson - blake.erickson@epfl.ch; Jonathan D. Adams - jonathan.adams@epfl.ch; Daniel J. Burns - danburns@mit.edu; Georg E. Fantner* - georg.fantner@epfl.ch

* Corresponding author

Further details on imaging and image processing

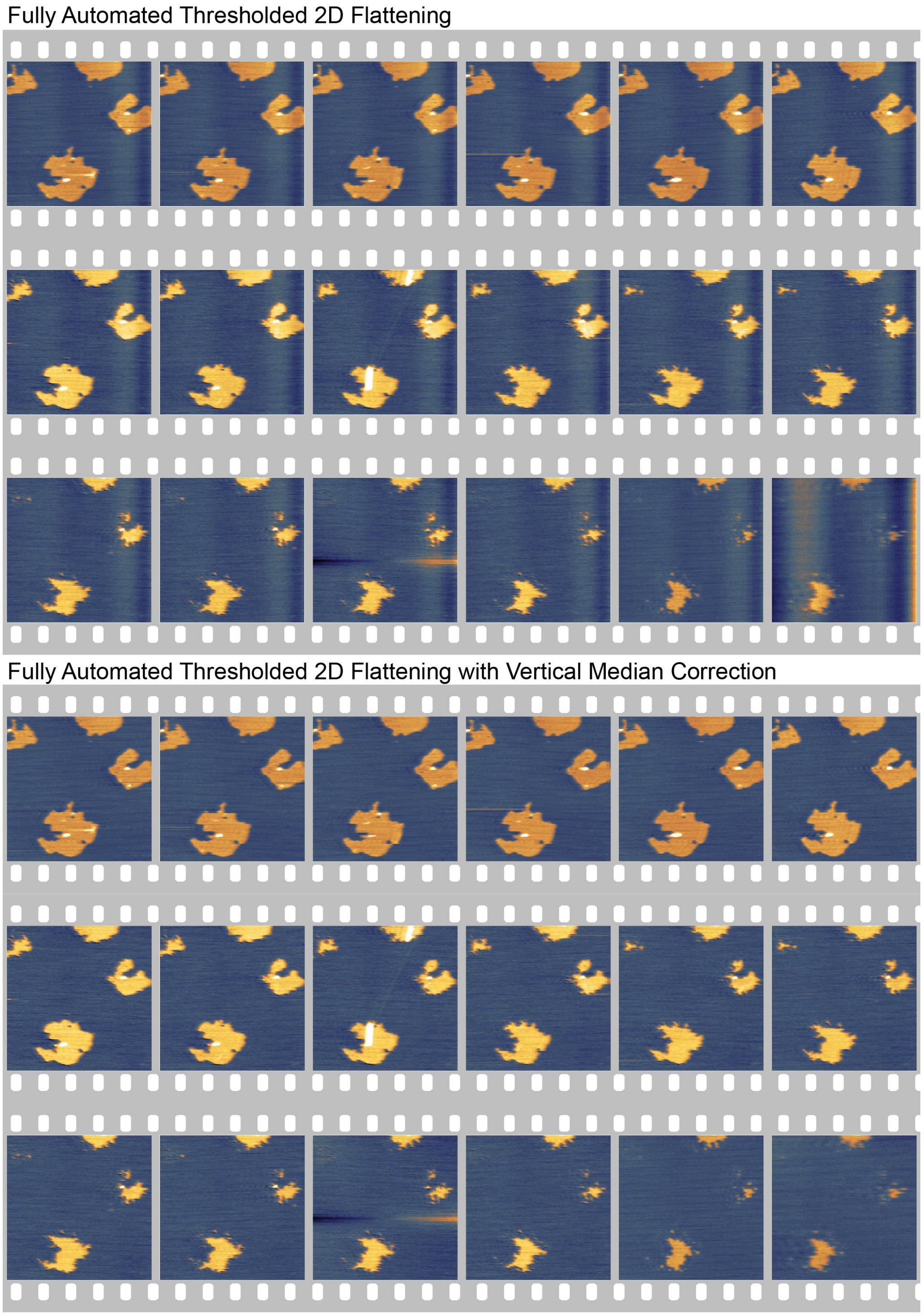

Figure S 1: This figure shows the entire image sequence from Figure 7. Each frame is approximately three seconds apart. The upper portion, Fully Automated thresholded 2-D Flattening, shows the default output of the algorithm. The lower portion, Fully Automated Thresholded 2-D Flattening with Vertical Median Correction, shows the results of an additional median correction in the left-right direction applied to each image. This vertical median correction is used to remove some residual distortions from turn-around ripple, which is a common error in HS-AFM. The vertical scale of all images is in nanometers.

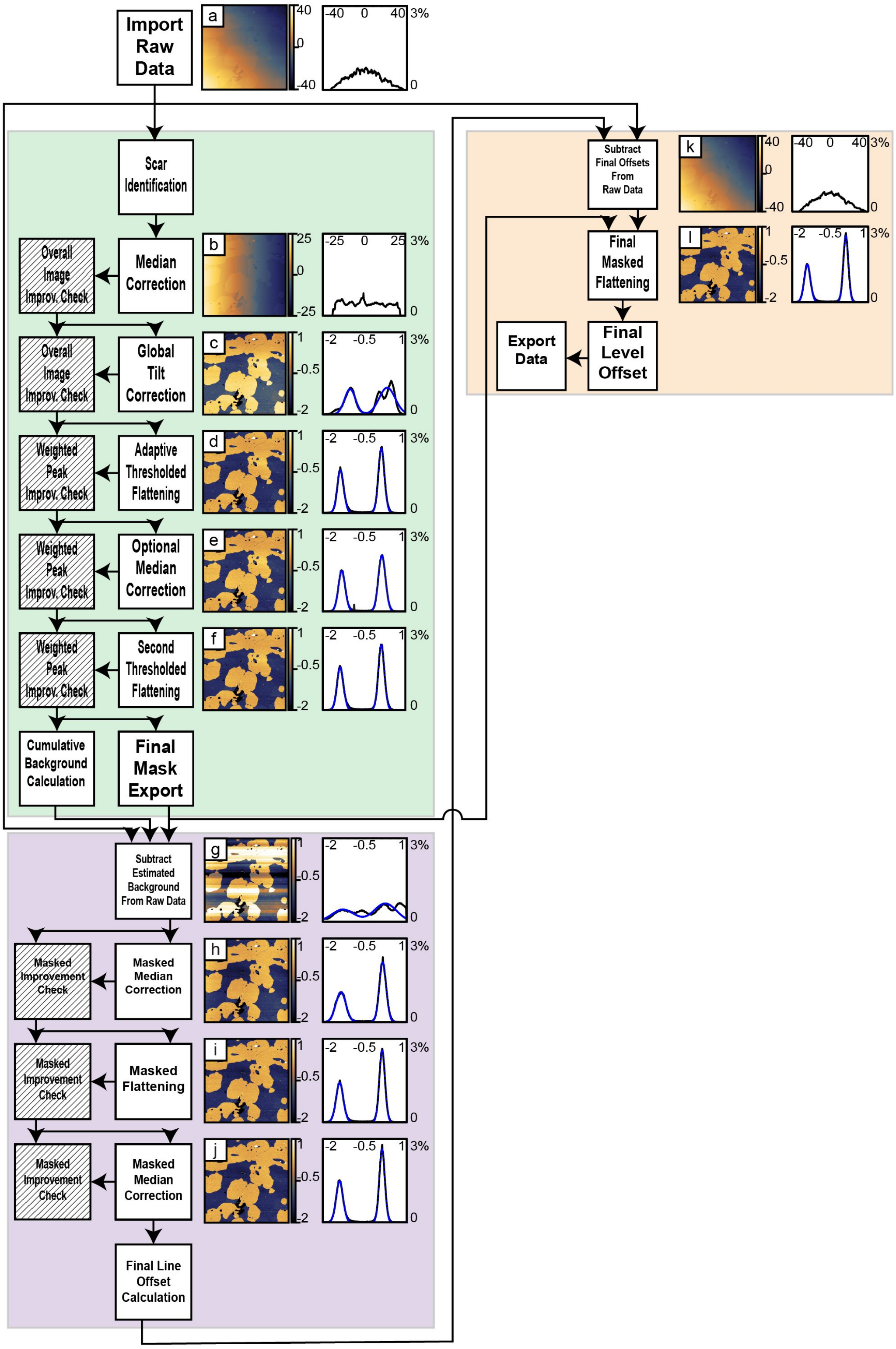

Figure S 2: This figure shows the complete flattening outline from start to finish. The background identification is shown in green. The 1-D offset determination is shown in purple and the final formatting and output is shown in peach. The vertical scale of all images and the horizontal scale of all histograms is in nanometers.

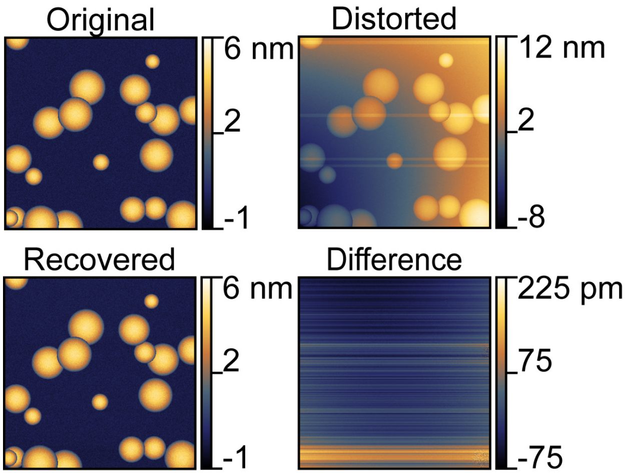

Figure S 3: This figure shows the algorithm's ability to correct the simulated distortion in Figure 1. The original image is the same as Figure 1B. The distorted image is the same as Figure 1E. The recovered image is the result of running the algorithm on Figure 1E. The difference image shows the recovered image subtracted from the original image. The difference image scale is within the noise of the starting image (Gaussian distribution with σ = 200 pm)

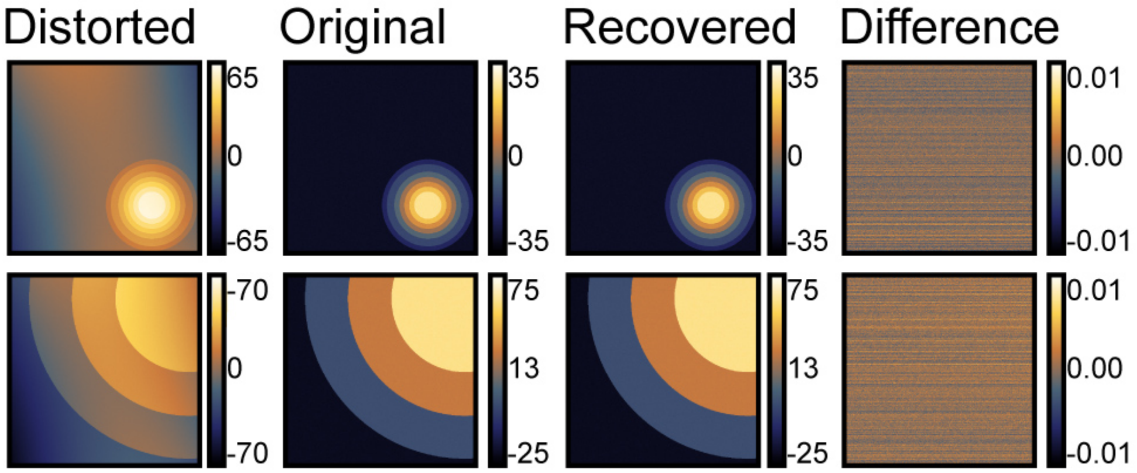

Figure S 4: This figure shows the results of the algorithm on a sample with multiple steps. The top row shows a sample with seven layers on top of a large background. The bottom row shows a sample with four distinct layers. The left most column shows the distorted image. The second column shows the original image. The third column shows the recovered image. Finally, the right most column shows the difference between the recovered image and the original image. In both cases the error (0.01 units) is much smaller than the noise level in the image (7.5 units).
