# Supplementary material for: Large-scale analysis of high-speed atomic force microscopy data sets using adaptive image processing
Source: Beilstein J Nanotechnol. 2012 Nov 13;3:747–58. doi: 10.3762/bjnano.3.84 (PMC3512124; doi:10.3762/bjnano.3.84)

Automated AFM Image Processing User Manual

Starting The Program

Open and run the "GUI_run_me.m" script in Matlab to start the program. The first thing to do is to select the folder that contains the images to be processed clicking on the "Change starting folder" button. Next, click the "Select images to process" button. At least 2 files must be selected. At this point the program reads the content of the selected files and displays the channels names in the panel "Channels to process." Select the channels of interest and then click on the "PARAMETERS" tab at the top. Default values are given for each parameter. These parameters need to be adapted for each sample type. After the parameters are updated, click on the "MAIN" followed by the "Process single image" to test the parameter values for flattening the first image.

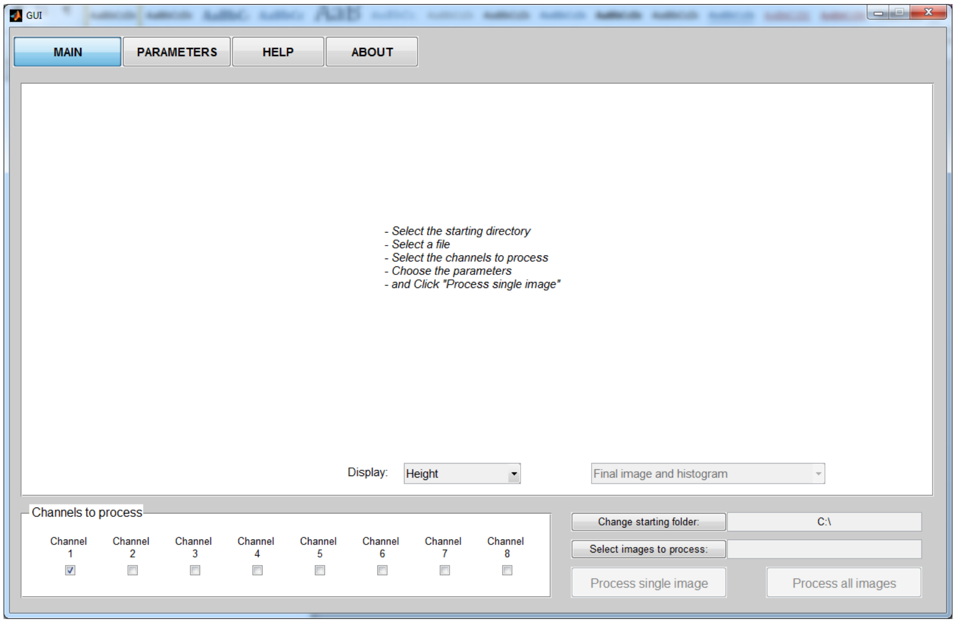

After the first image is processed, the processed image and corresponding histogram are displayed. A popup menu allows the user to change the displayed channel. It is also possible to display the different steps of the image processing using the popup designed for this purpose.

Example:

After the First Median Correction:

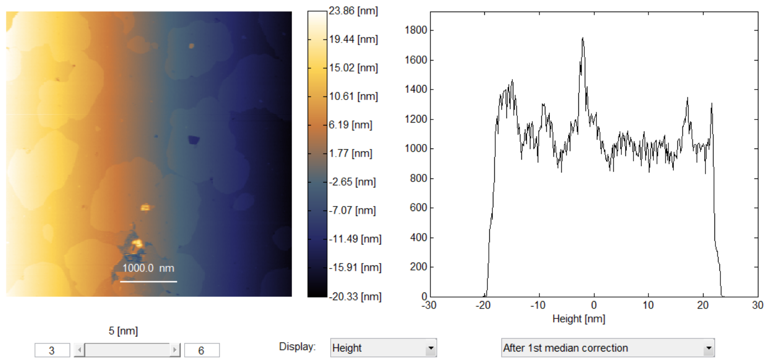

Final Image:

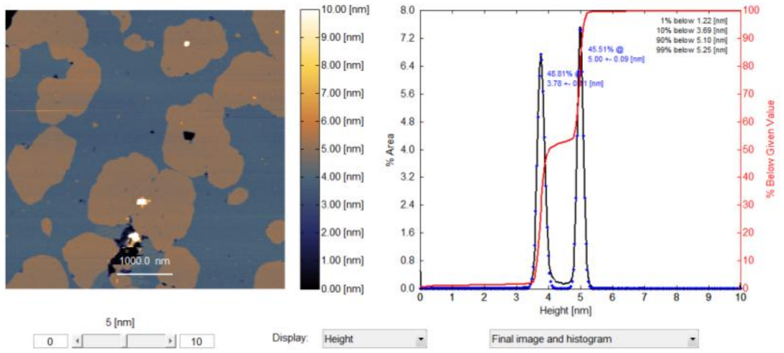

The settings can then be adjusted and a single image can be processed as many times as needed in order to obtain a properly flattened final image. Careful setting of the parameters will give better results in the long run.

Once the right parameters have been found, all the selected images can be processed by clicking on the "Process all images" button. The histograms, gray scale images without markup and color images with markup are all saved as .fig and .jpg files in a subfolder named "Matlab."

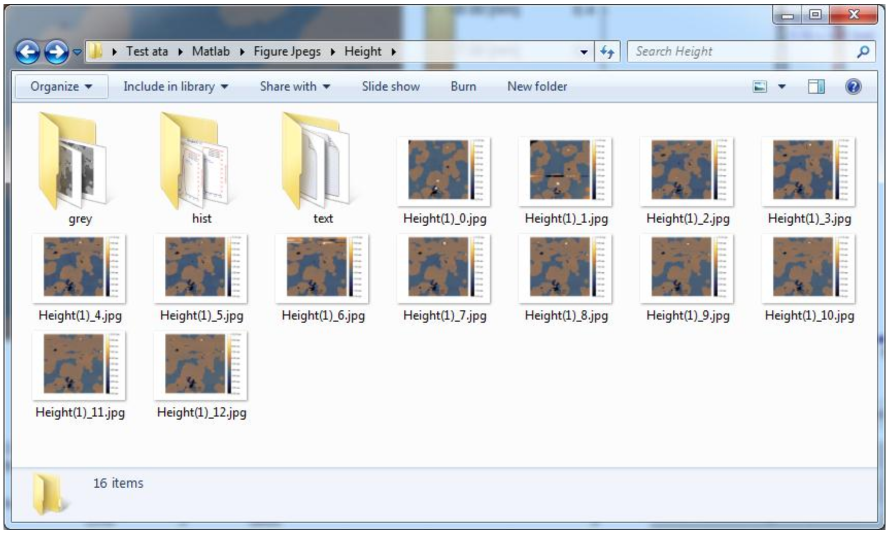

Once a good set of processing parameters is found for a given sample, they can be saved for future use under the “PARAMETERS” tab. The saved settings can then be reused later by clicking the Load button.

Choosing the Parameters

The parameters tab contains two main panels: one for the general settings, and one for the parameters per channel. Only the parameters for the channels to be processed are enabled.

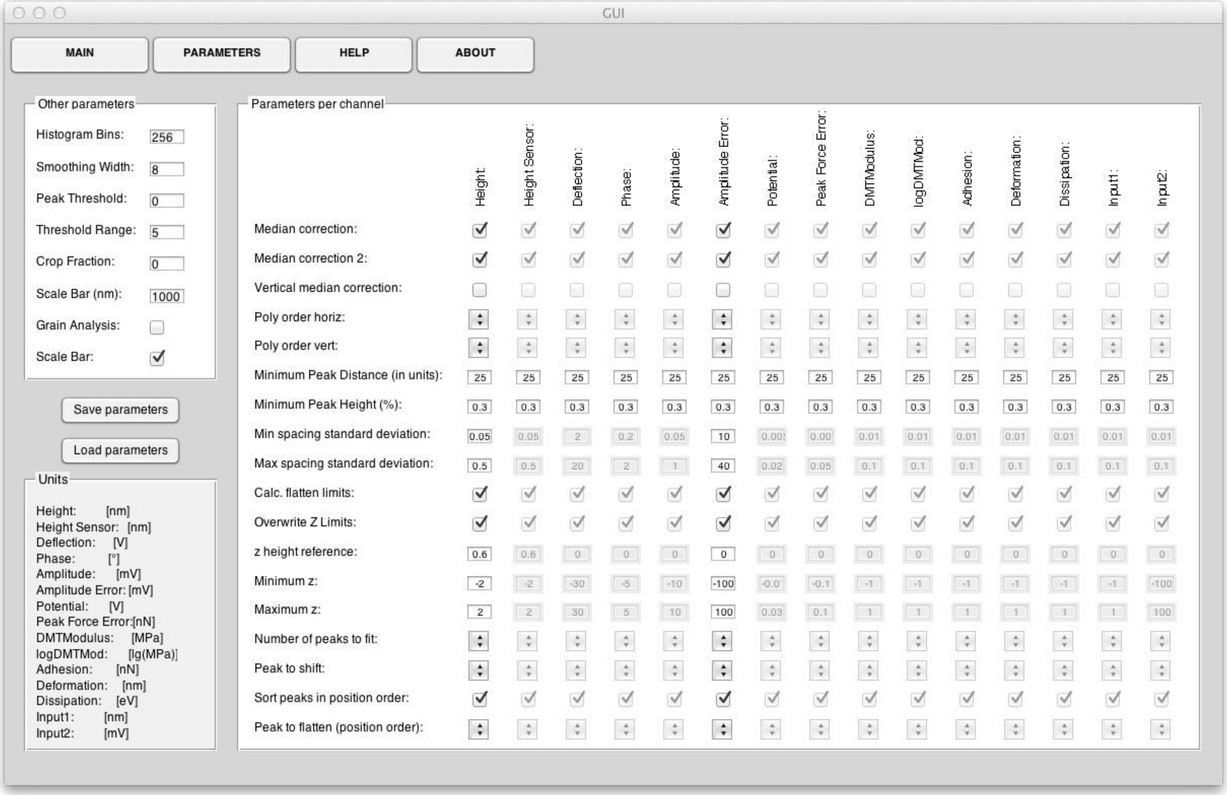

Global Parameters

Parameter	Explanation
Histogram Bins	The values in the image will be distributed in a number of bins equal to histogram bins. The default value is 256.
Smoothing Width	The histogram is smoothed using the Loess method to remove minor peaks. Histogram smoothing level sets the span. Example (histogram smoothing level = 5): 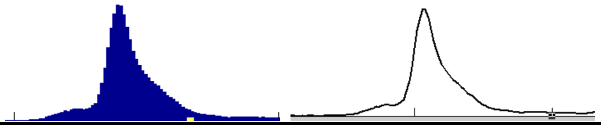
Peak Threshold	Peak threshold is the minimum height difference between a peak and its neighboring values. Only the peaks that exceed their neighbors by at least peak threshold are found.
Threshold Range	Sets the search range for the thresholding as a multiple of the standard deviation of the fitted peak. Bigger values include more data. The recommended range is between 2 and 5.
Crop Fraction	Crops an image prior to processing from the outer edge towards the center. 0% = no cropping. 50% keeps the middle 50% only. 100% would crop out all data.
Grain Analysis	Turns on optional particle detection. This section is not supported.
Scale Bar (input box)	Sets the length of the scale bar in nm
Scale Bar (check box)	Turns on or off the scale bars in the final image

Parameters per Channel

<table>
<tr><th>Parameter</th><th>Explanation</th></tr>
<tr><td>Median correction</td><td>If checked, applies a line match correction with scar correction at the beginning.</td></tr>
<tr><td>Median correction 2</td><td>If checked, applies a second line match correction after the first median correction, a global tilt correction and the first adaptive thresholded flattening (polynomial surface fit and removal).</td></tr>
<tr><td>Vertical median correction</td><td>If checked, applies a vertical line match correction after all other processing steps to just the masked data. This is experimental, and meant for HS-AFM resonance artifacts only.</td></tr>
<tr><td>Poly order horiz:
and
Poly order vert:</td><td>Parameters for the adaptive image thresholding algorithm and background removal. The data is fit to a polynomial surface of order given by Poly order horiz and Poly order vert. The extrapolated background is then subtracted from the whole image.</td></tr>
<tr><td>Minimum peak height (%)</td><td>When searching for peaks in the histogram, only peaks that exceed minimum peak height are found. Example (Minimum peak height = 0.3%). Looking at the intermediate steps can help set the proper value for this parameter.
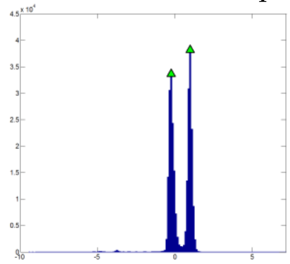</td></tr>
<tr><td>Minimum Peak Distance (in units of a given channel)</td><td>When searching for peaks in the histogram, only peaks that are separated by Minimum Peak Distance are returned. This allows ignoring small peaks occurring in the neighborhood of a larger peak. This parameter is very important, as demonstrated in the figure below. The identified peaks may be very different according to the value of Minimum Peak Distance.
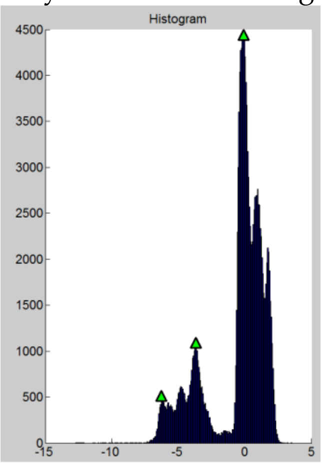
</td></tr>
<tr><td>Min spacing standard</td><td>During the fitting of the data by a summation of Gaussians, Min spacing</td></tr>
</table>

deviation and *Max spacing standard deviation*	*standard deviation* and *Max spacing standard deviation* are used to determine the fitting bounds (lower and upper limits as well as starting value for the standard deviation). This can be useful to set manually if the initial image is very distorted and the automatic parameter generation is not restricting the values enough.
Calc. flatten limits	If checked, the two previous parameters (*Min spacing standard deviation and Max spacing standard deviation*) are estimated from peaks in the intermediate images and the user input values are ignored.
Overwrite Z limits	If checked, the minimum and maximum display values are the ones defined by the user. If not, the program calculates the minimum and maximum values.
z height reference	This value shifts the center of the *Peak to shift* to a user defined reference value. This is useful if a sample is of a known thickness, or to ensure consistency with other data sets for presentation.
Minimum Z and *Maximum Z*	These two parameters set the display range for the image and histogram if *Overwrite Z limits* is checked. An example is shown on next page.
Number of peaks to fit	This parameter sets the number of peaks to fit. This parameter should correspond the number of distinct levels in the image.
Peak to shift	This value determines the peak that will be offset by *z height reference*.
Sort peaks in position order	This parameter allows sorting the peaks either in position order (if checked) or in amplitude order (if not checked). This parameter should generally remain checked.
Peak to flatten (position order)	This gives the peak to be flattened for each channel. If *sort peaks in position order* is checked, *Peak to be flattened* = n will flatten the peak at the nth position, and if not checked, it will flatten the peak with the nth amplitude (descending).

The minimum and maximum display values as well as the reference z height can be directly changed using the slider and the two edit boxes under the image. This will also change their values in the Parameters tab.

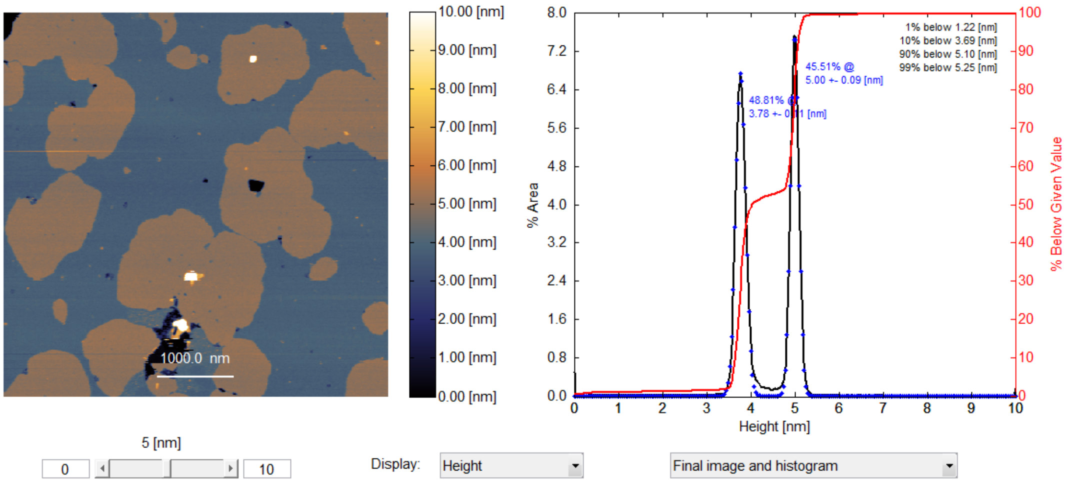

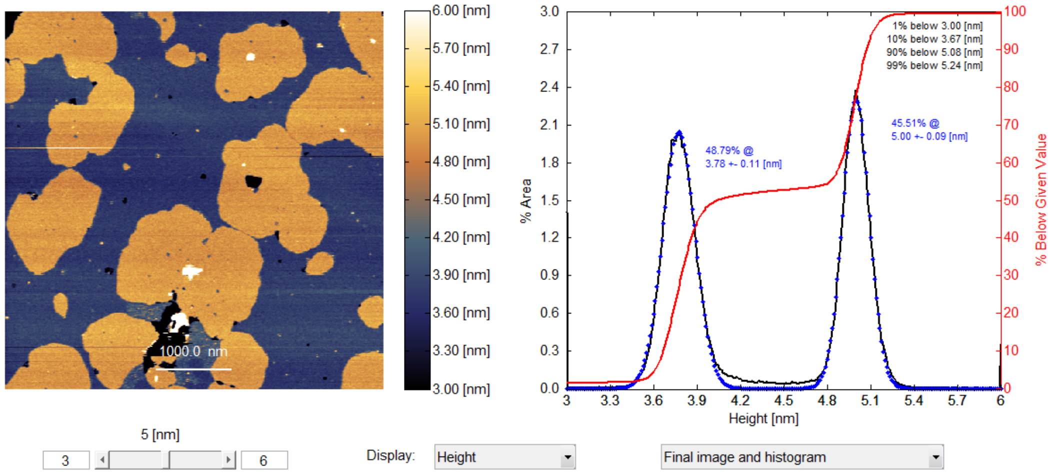

Supplement: File 3 — User Manual The user manual presented here contains a brief description of how to use the program and the parameters available for each channel. [file Beilstein_J_Nanotechnol-03-747-s003.pdf]
